# Loss of Primary Cilia Protein IFT20 Dysregulates Lymphatic Vessel Patterning in Development and Inflammation

**DOI:** 10.3389/fcell.2021.672625

**Published:** 2021-05-14

**Authors:** Delayna Paulson, Rebecca Harms, Cody Ward, Mackenzie Latterell, Gregory J. Pazour, Darci M. Fink

**Affiliations:** ^1^Department of Chemistry and Biochemistry, South Dakota State University, Brookings, SD, United States; ^2^BioSNTR, South Dakota State University, Brookings, SD, United States; ^3^Program in Molecular Medicine, University of Massachusetts Medical School, Worcester, MA, United States

**Keywords:** primary cilia, lymphatic, lymphangiogenesis, lymphatic development, corneal inflammation, IFT20, vascular patterning

## Abstract

Microenvironmental signals produced during development or inflammation stimulate lymphatic endothelial cells to undergo lymphangiogenesis, in which they sprout, proliferate, and migrate to expand the vascular network. Many cell types detect changes in extracellular conditions via primary cilia, microtubule-based cellular protrusions that house specialized membrane receptors and signaling complexes. Primary cilia are critical for receipt of extracellular cues from both ligand-receptor pathways and physical forces such as fluid shear stress. Here, we report the presence of primary cilia on immortalized mouse and primary adult human dermal lymphatic endothelial cells *in vitro* and on both luminal and abluminal domains of mouse corneal, skin, and mesenteric lymphatic vessels *in vivo*. The purpose of this study was to determine the effects of disrupting primary cilia on lymphatic vessel patterning during development and inflammation. Intraflagellar transport protein 20 (IFT20) is part of the transport machinery required for ciliary assembly and function. To disrupt primary ciliary signaling, we generated global and lymphatic endothelium-specific IFT20 knockout mouse models and used immunofluorescence microscopy to quantify changes in lymphatic vessel patterning at E16.5 and in adult suture-mediated corneal lymphangiogenesis. Loss of IFT20 during development resulted in edema, increased and more variable lymphatic vessel caliber and branching, as well as red blood cell-filled lymphatics. We used a corneal suture model to determine ciliation status of lymphatic vessels during acute, recurrent, and tumor-associated inflammatory reactions and wound healing. Primary cilia were present on corneal lymphatics during all of the mechanistically distinct lymphatic patterning events of the model and assembled on lymphatic endothelial cells residing at the limbus, stalk, and vessel tip. Lymphatic-specific deletion of IFT20 cell-autonomously exacerbated acute corneal lymphangiogenesis resulting in increased lymphatic vessel density and branching. These data are the first functional studies of primary cilia on lymphatic endothelial cells and reveal a new dimension in regulation of lymphatic vascular biology.

## Introduction

Regulation of lymphatic vessel patterning is critical for the establishment of lymphatic vascular networks during development and pathological tissue remodeling events such as inflammation and wound healing. Lymphatic expansion is driven by a set of cellular mechanisms that activate and mobilize individual endothelial cells residing in a pre-existing vessel to sprout, proliferate, undergo directional chemotaxis, and remodel intercellular junctions to form new vessels. Properly patterned lymphatic vessels govern tissue fluid homeostasis and transport soluble antigens and immune cells to draining lymph nodes. During development, lymphatic patterning begins at approximately E9.5 when COUPTFII and SOX18 upregulate expression of PROX1 in venous endothelial cells of the cardinal vein to establish the first lymphatic endothelial cells (LECs; Wigle and Oliver, 1999; Petrova et al., 2002; François et al., 2008; Johnson et al., 2008; Srinivasan et al., 2010; Hägerling et al., 2013). These cells form lymph sacs, which expand to form lymphatic vessels separated from the blood circulation by lymphovenous valves (Cha et al., 2016; Geng et al., 2016). Further proliferation, branching, and maturation establishes a network of lymphatic vasculature throughout the embryo consisting of blind-ended capillaries that coalesce into lymphatic collecting vessels invested with smooth muscle cells and containing intraluminal valves (Lutter et al., 2012; Scallan et al., 2016). Inflammation activates existing lymphatic capillaries to undergo lymphangiogenesis, thus increasing capacity to transport edematous fluid and support egress of antigen presenting cells and lymphocytes out of inflamed tissue (Baluk et al., 2005; Maruyama et al., 2005; Pflicke and Sixt, 2009; Tal et al., 2011).

Lymphatic vessel patterning and function are dysregulated and contribute to disease pathology in many conditions, including chronic inflammation, lymphedema, and malignancy (Skobe et al., 2001; Schwager and Detmar, 2019; Oliver et al., 2020). The canonical mechanism governing both developmental and inflammation-associated lymphangiogenesis is VEGF-C stimulating VEGFR-2/3 on LECs, along with co-receptor NRP-2 (Karkkainen et al., 2004; Haiko et al., 2008; Kataru et al., 2009; Xu et al., 2010; Kelley et al., 2013). Other factors induce lymphangiogenesis in specific contexts including angiopoietins (Gale et al., 2002; Morisada et al., 2005; Tammela et al., 2005; Dellinger et al., 2008), TNF-α(Ji et al., 2014), FGF-2 (Cao et al., 2012), IGF1/2 (Björndahl et al., 2005), EGF (Marino et al., 2013), HGF (Cao et al., 2006), PDGF-BB (Cao et al., 2004), and NGF (Fink et al., 2014). Despite the identification of these lymphangiogenic factors, the mechanisms governing lymphatic vessel patterning are not fully understood. This is particularly evident in studies of viral infection (Loo et al., 2017), lymphatic vessel regression (Baluk et al., 2005; Cursiefen et al., 2006; Kelley et al., 2011), and chronic inflammation (Kelley et al., 2013) in which lymphatic vessel remodeling events are highly variable and do not follow current paradigms. This evidence suggests that additional unknown elements regulate lymphatic vessel patterning.

Primary cilia are non-motile sensory organelles that govern proliferation, migration, cell polarity, and fluid shear stress sensing in other cell types, including blood endothelial cells. Primary cilia house receptor-ligand signaling pathways such as Hedgehog (Huangfu et al., 2003; Huangfu and Anderson, 2005; Clement et al., 2009), Wnt (Jonassen et al., 2008; McDermott et al., 2010), Notch (Ezratty et al., 2011; Grisanti et al., 2016; Chen et al., 2017), PDGFRα (Schneider et al., 2005; Noda et al., 2016; Schmid et al., 2018), and TGF-β (Clement et al., 2013), as well as mechanosensation machinery (Praetorius and Spring, 2001), such as polycystins PKD1/2 (Pazour et al., 2002; Nauli et al., 2008). Disruption of primary cilia signaling dysregulates formation of lumenized structures such as kidney tubules (Pazour et al., 2000; Jonassen et al., 2008) and breast ducts (McDermott et al., 2010), and causes cardiovascular disease (Pala et al., 2018). Studies across *in vivo* systems including zebrafish and mice have established that disruption of pathways known to function via primary cilia in other cell types dysregulates lymphangiogenesis (James et al., 2013; Murtomaki et al., 2013; Coxam et al., 2014; Fatima et al., 2014; Outeda et al., 2014; Huang et al., 2015; Betterman et al., 2020). A single previous report (Bailey et al., 2009) identified acetylated α-tubulin+ structures on LYVE-1+ cells in thin sections of pancreatic ductal adenocarcinoma, suggesting that primary cilia may modulate tumor-stroma interactions in the tumor microenvironment. When and how LECs express primary cilia across tissues, developmental stages, inflammation states, and vessel domains is not known. The purpose of this study was to determine if and when primary cilia are present on mammalian LECs and how they regulate lymphatic vessel patterning.

We used immunofluorescence microscopy to demonstrate that LECs possess primary cilia both *in vitro* and *in vivo* across diverse microenvironmental conditions. Based on primary cilia function in other cell types, we hypothesized that primary cilia on LECs regulate cellular behaviors important for lymphangiogenesis. To evaluate how loss of primary cilia impacts lymphatic vessel patterning, we knocked out intraflagellar transport protein 20 (IFT20), a protein critical for primary cilia assembly and ciliary cargo transport, and assessed lymphangiogenesis during mouse development and corneal inflammation. This paper presents the primary cilium as a novel LEC organelle with regulatory functions during lymphatic vessel patterning and discusses the implications of primary cilia signaling for fundamental lymphatic endothelial biology.

## Materials and Methods

### Lymphatic Endothelial Cell Culture and Immunofluorescence

Immortalized mouse mesenteric lymphatic endothelial cells (SVLECs) were a kind gift from J. Steven Alexander (Ando et al., 2005) (Louisiana State University Health Shreveport) and were routinely cultured with Dulbecco’s Modified Eagle’s Medium (Corning, 10-017-CV) supplemented with 10% fetal bovine serum (Thermo Fisher Scientific) and penicillin/streptomycin (Thermo Fisher Scientific) at 37°C and 5% CO_2_. Primary human dermal lymphatic endothelial cells (HDLECs) obtained from PromoCell (c-12217) were routinely cultured with Endothelial Cell Growth Medium MV 2 (PromoCell, c-22022) supplemented with the corresponding SupplementMix (PromoCell, c-39226) at 37°C and 5% CO_2_. Under serum starvation protocols, SVLECs and HDLECs were cultured with 0 and 0.1% serum at 37°C and 5% CO_2_ for 24 and 48 h, respectively. For immunofluorescence staining protocols, SVLECs were seeded on sterile poly-L-lysine coated coverslips (Corning), and HDLECs were seeded on sterile uncoated coverslips (Thermo Fisher Scientific). Both SVLECs and HDLECs were fixed in chilled methanol for 20 min. Cells were then blocked and permeabilized in a solution containing 0.15% Triton-X 100 and 1% bovine serum albumen (BSA) in 1× PBS. Primary antibodies were incubated for one hour at room temperature (RT) in the blocking solution followed by PBS washes. Secondary antibodies were incubated for 45 min at RT in the blocking solution followed by PBS washes. Coverslips were mounted in Fluoromount-G with DAPI (Thermo Fisher Scientific, 00-4959-52) and imaged by epifluorescence microscopy.

### Mice

All experimental procedures were approved by the Institutional Animal Care and Use Committees of South Dakota State University or the University of Massachusetts Medical School. C57BL/6J (Stock# 000664), Prox1CreERT2 (*Prox1^TM 3(cre/ERT2)Gco^*/J, Stock# 022075), and Lyve1Cre (B6;129P2-*Lyve1^TM 1.1(EGFP/cre)Cys^*/J, Stock# 012601) mice were purchased from Jackson Laboratories. Lyve1CreERT2 (Connor et al., 2016) mice were a kind gift from Dr. Richard M. Tempero (Boys Town National Research Hospital, Omaha, NE, United States). Development of *Ift20*^*fl/fl*^ mice was described previously (Jonassen et al., 2008). Development of global and LEC-specific IFT20 knockout (KO) mice is described below. Development of Lyve1CreERT2;tdTomato mice with LEC-specific expression of tdTomato reporter protein was described previously (Connor et al., 2016). Adult mice were euthanized by ketamine/xylazine overdose or CO_2_ inhalation and cervical dislocation. Embryos were collected following euthanasia of pregnant dams. Mice were genotyped by standard PCR methods.

### Development of Global and LEC-Specific IFT20 KO Mice

*Ift20*^*fl/fl*^ females were bred to *Ift20*^*null/+*^;CAGG-CreER^TM^ (Hayashi and McMahon, 2001) males. E0.5 was designated as noon on the day of plug identification. CAGG-CreER^TM^ recombinase activity was induced in pregnant dams at E7.5 by oral gavage of tamoxifen suspended in vegetable oil (100 μL, 10 mg/mL). Embryos were harvested at E16.5 and fixed in 10% formalin overnight at 4°C. Following fixation, embryos were rinsed with PBS and stored in 70% ethanol.

Lymphatic endothelial cell-specific IFT20 KO strains were generated by crossing *Ift20*^*fl/fl*^ mice with Cre-positive mice (Prox1CreERT2, Lyve1CreERT2, or Lyve1Cre). Cre-positive progeny of this cross bearing a single floxed *Ift20* allele were then crossed with *Ift20*^*fl/fl*^ mice to generate the desired experimental *Ift20*^*fl/fl*^;Cre-positive genotype. Littermates of mixed genotypes were included in control groups, including mice that were Cre-positive or Cre-negative and heterozygous or wild type for floxed *Ift20*. For CreERT2 lines, 4-hydroxytamoxifen (Sigma-Aldrich, H7904-25MG) was suspended in sunflower seed oil and injected intraperitoneally on three consecutive days (200 μL, 5 mg/mL).

### Corneal Inflammation Assays

The corneal model of suture-mediated inflammation, wound recovery, and recurrent inflammation was previously described (Kelley et al., 2011, 2013; Fink et al., 2014). Briefly, we placed four 10-0 prolene monofilament sutures into the healthy mouse cornea and left them in place for 7 days to induce inflammation. Sutures were removed on day 7 and corneas healed for 14 days. To induce recurrent inflammation, we placed four sutures into corneas after wound healing (day 21) and left them in place for 7 days. Homeostasis control corneas were harvested from unmanipulated mice. Surgery was performed under general (ketamine 100 mg/kg, xylazine 10 mg/kg) and topical (proparacaine hydrochloride, USP, 0.5%) anesthesia. Pain was controlled with slow-release buprenorphine (0.1 mg/kg, Zoopharm), and subcutaneous saline was administered to prevent dehydration. Gentamicin ophthalmic ointment (USP, 0.3%) was applied post-operatively.

### Corneal Tumor Model

B16-F10 mouse melanoma cells were obtained from ATCC (CRL-6475) and routinely cultured with Dulbecco’s Modified Eagle’s Medium (Corning, 10-017-CV) supplemented with 10% fetal bovine serum (Thermo Fisher Scientific) and penicillin/streptomycin (Thermo Fisher Scientific) at 37°C and 5% CO_2_. To model a tumor microenvironment, B16-F10 cells were injected into healthy Lyve1CreERT2;tdTomato mouse corneas on the same day as suturing with four 10-0 prolene monofilament sutures. Injections were performed under general and topical anesthesia and pain was controlled as described above. Lymphatic vessel growth was observed by intravital imaging using a Zeiss SteREO Discovery.V8 fluorescence stereomicroscope on day 7 (data not shown). Corneas were harvested on day 8.

### Whole-Mount Tissue Immunofluorescence

The following mouse tissues were immunostained and imaged as whole-mounts: adult ear, adult cornea, adult mesentery, E16.5 skin, and E16.5 mesentery. Adult mouse ears were dissected to separate ventral and dorsal halves, then fixed in 4% PFA at RT for 2 h. Adult mouse eyes were enucleated and fixed in 1% PFA for 1 h at RT. The cornea was then dissected out and fixed in 1% PFA for an additional hour at RT. Adult mouse mesentery samples were dissected onto a supportive plastic mesh and fixed in 4% PFA at RT for 1 h. E16.5 IFT20 KO embryos were fixed overnight in 10% formalin. Skin and mesentery were dissected from IFT20 KO embryos for whole-mount staining. Following fixation, whole-mount tissue samples were rehydrated in 1X PBS for one hour at RT and blocked and permeabilized for one hour at RT in PBS++, a solution containing 5.2% BSA, 0.3% Triton X-100, and 0.2% sodium azide in 1X PBS at pH 7.4. Primary and secondary antibodies were applied in PBS++ overnight at RT. Following primary and secondary antibody incubations, three 1 h washes were completed in PBS+, a solution containing 0.2% BSA, 0.3% Triton X-100, and 0.2% sodium azide in 1X PBS at pH 7.4. Samples were mounted in Fluoromount-G without DAPI (Thermo Fisher Scientific, 00-4958-02).

### Antibodies

Primary antibodies used in cell and tissue immunofluorescence include: rabbit anti-mouse LYVE-1 1:200 (abcam, ab33682), goat anti-human PROX-1 1:200 (R&D Systems, AF2727), mouse anti-mouse ARL13B 1:200 and 1:4,000 (Neuromab, 75-287), rabbit anti-mouse IFT20 1:1,000 (Proteintech, 13615-1-AP), goat anti-mouse NRP-2 1:200 (R&D Systems, AF567), goat anti-human podocalyxin 1:100 (R&D Systems, AF1658), goat anti-mouse integrin α-9 1:100 (R&D Systems, AF3827), rat anti-mouse LYVE-1 1:50 (Santa Cruz, sc-65647), and rat anti-mouse pHH3 1:100 (Sigma Aldrich, H9908). Directly conjugated primary antibodies used in whole-mount tissue immunofluorescence include: mouse anti-mouse 611B1-AF647 1:500 (Santa Cruz, sc-23950 AF647) and mouse anti-mouse smooth muscle actin-Cy3 1:300 (Sigma-Aldrich, C6198). Secondary antibodies used in cell and tissue immunofluorescence at concentrations of 1:10,000 and 1:500, respectively, include: goat anti-mouse 488, donkey anti-rabbit 488, goat anti-mouse 555, chicken anti-goat 647 (Invitrogen), donkey anti-rabbit 555 (abcam), donkey anti-rat 594 (Thermo Fisher Scientific), chicken anti-goat 488 (Thermo Fisher Scientific), goat anti-rat 550 (Thermo Fisher Scientific), and goat anti-rat 647 (Thermo Fisher Scientific). Primary antibodies used for Western blot analysis include: rabbit anti-mouse/human PROX-1 1:1,000 (abcam, ab101851) and mouse anti-mouse β-actin 1:1,000 (Santa Cruz, sc-47778). Secondary antibodies include: donkey anti-mouse IRDye 800CW 1:15,000 (Licor, 926-32212) and donkey anti-rabbit IRDye 680RD 1:15,000 (Licor, 926-68073).

### Microscopy

Epifluorescence microscopy was performed using a DMI 4000 B microscope (Leica) with a KUBler CODIX EL6000 light source (Leica) and a Zyla 4.2 sCMOS camera (ANDOR). Two photon microscopy was performed using a FLUOVIEW FVMPE-RS Multi Photon Laser Scanning Microscope (Olympus) and InSight and MaiTai tunable lasers (SpectraPhysics). Laser scanning confocal microscopy was performed using a FLUOVIEW FV1200 scanning confocal microscope (Olympus) interfaced with an IX81 microscope (Olympus) using 488, 559, and 635 laser lines. A spinning disk confocal microscope with fully automated iMIC platform (Till Photonics), four lasers (445, 515, 561, and 645nm), and a CMOS camera was used to image whole-mount corneas. A Zeiss Stemi 305 compact stereo dissecting microscope with a 1080P HP digital video camera was used to image intact embryos and perform surgery and dissections. A Zeiss SteREO Discovery.V8 fluorescence stereomicroscope and a Zeiss Axio Zoom.V16 fluorescence stereo zoom microscope with Apotome2 structured illumination module and Axiocam 503 monochrome cameras were used to image tissues and perform surgery. Z-slice thickness from confocal and two photon microscopy is indicated in figure legends.

### Image Analysis

**FIGURE 1:**
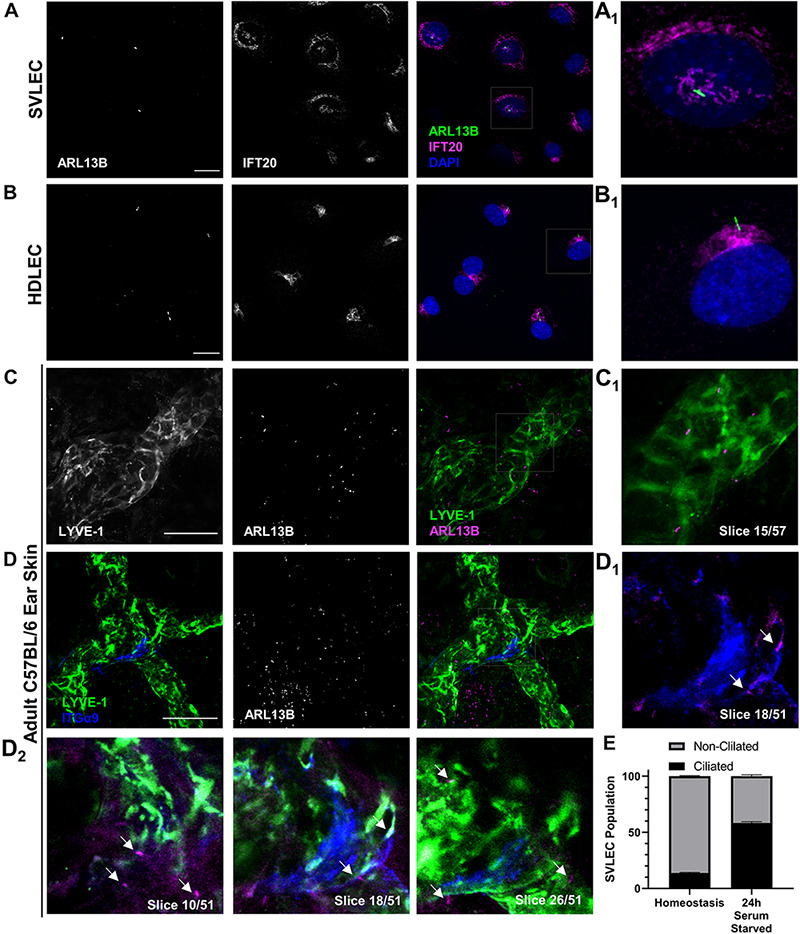
PC assemble on LECs both *in vitro* and *in vivo*. **(A,B)** Epifluorescence microscopy of serum starved SVLECs **(A)** and HDLECs **(B)** immunostained with antibodies against ARL13B and IFT20, and nuclei labeled with DAPI reveals primary cilia on immortalized mouse and primary human LECs *in vitro*. **(A_1_,B_1_)** Single cell from panels **(A,B)**. **(C,D)** Maximum intensity projections (MIPs) from laser scanning confocal z-stacks of adult C57BL/6J whole mount ear skin immunostained with antibodies against LYVE-1, ARL13B, and ITGα9 reveal primary cilia on both luminal and abluminal surfaces of lymphatic vessels *in vivo*. **(C_1_)** A single 1 μm z-slice shows abluminal primary cilia on a LYVE-1 positive lymphatic vessel. **(D_1__–__2_)** Intraluminal primary cilia are observed near lymphatic valves identified by ITGα9. Arrows indicate both luminal and abluminal primary cilia. **(E)** Quantification of **(A)** represented as an average of three biological replicates. Each biological replicate included at least three fields of view from each of at least two technical replicates. **(C,D)** Images are representative from three animals. **(A,B)** Scale bar = 20 μm. **(C,D)** Scale bar = 50 μm.

**FIGURE 2:**
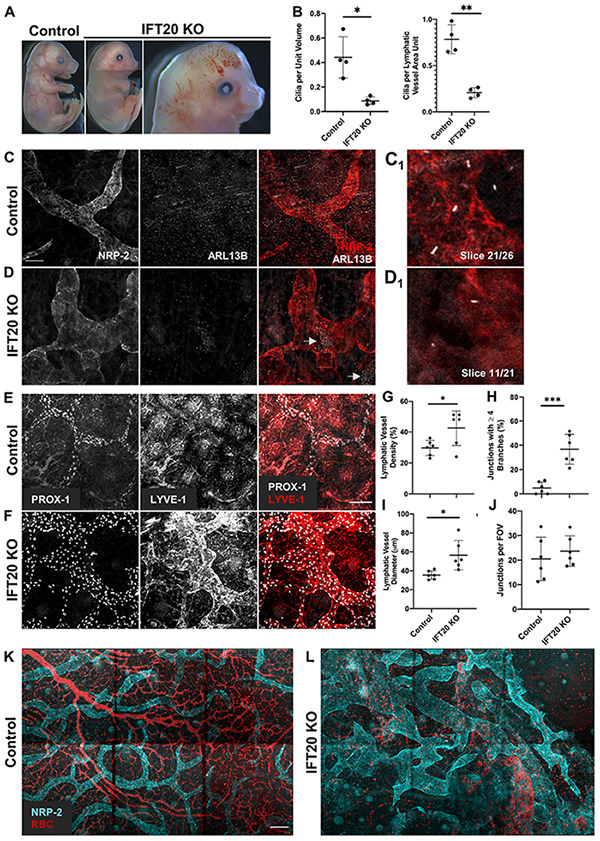
Global IFT20 KO mice show severe lymphatic vessel patterning defects at E16.5. **(A)** Administration of tamoxifen to pregnant dams at E7.5 causes polydactyly, craniofacial abnormalities, hydrops, and abnormal vasculature with gross hemorrhaging in IFT20 KO embryos at E16.5 vs. control. Images of entire embryo were generated by aligning separate images of head, abdomen, and rump. **(B)** Quantification of panels **(C,D)** where “unit volume” represents 300 μm^3^ of tissue, approximating the volume of a single cell, and “LV area unit” represents 75 μm^2^ of lymphatic vessel. Each data point represents a per embryo average of at least 2 fields of view and is from 4 control and 4 KO animals across 3 litters. **(C,D)** MIPs from laser scanning confocal z-stacks of whole mount skin immunostained with antibodies against neuropilin-2 (NRP-2) and ARL13B. Arrows in panel **(D)** indicate autofluorescent red blood cells in lymphatic vessel lumens. **(C_1_,D_1_)** Single 1 μm z-slices showing primary cilia on the abluminal surface of NRP-2 positive lymphatic vessels. **(E,F)** MIPs from multiphoton z-stacks of full thickness skin samples immunostained with antibodies against LYVE-1 and PROX-1 show malformed lymphatic vasculature of significantly greater vessel density, diameter, and branching in the IFT20 KO **(F)** vs. control **(E)**. **(G–J)** Quantification of panels **(E,F)** where each data point represents a per embryo average of at least 3 fields of view and is from 6 KO and 6 control embryos across 4 litters. **(K,L)** MIPs from tiled multiphoton z-stacks of full thickness skin samples immunostained with antibodies for NRP-2 further demonstrate lymphatic patterning defects in the mutant **(L)** vs. control **(K)**. Autofluorescent red blood cells are shown in red and suggest hemorrhaging in the IFT20 KO (**L**). Scale bars = 50 μm. ^∗∗∗^*p* < 0.0001; ^∗∗^*p* < 0.005; ^∗^*p* < 0.05.

**FIGURE 3:**
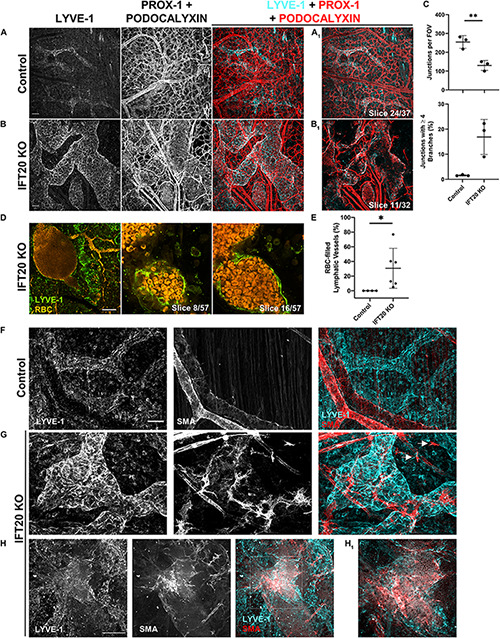
Lymphatic vessels in E16.5 IFT20 KO skin have intraluminal red blood cells and aberrant SMA+ cell coverage. **(A,B)** MIPs from laser scanning confocal z-stacks from whole mount control **(A)** and IFT20 KO **(B)** skin immunostained with antibodies against LYVE-1, PROX-1, and podocalyxin, a sialoglycoprotein expressed in both blood and lymphatic endothelium. **(A_1_,B_1_)** Single 1 μm z-slices. **(C)** Quantification of blood vessel junctions from panels **(A,B)** where each data point represents a per embryo average of at least 2 fields of view and is from 3 KO and 3 control embryos. **(D)** MIP and single 1 μm z-slices from two photon z-stacks of whole-mount skin immunostained for LYVE-1. Autofluorescent RBCs are shown in orange. **(E)** Quantification of RBC-filled lymphatics from whole-mount skin immunostained for NRP-2 (as in Figures 2C,D), where each data point represents a per embryo average of at least 4 fields of view and is from 4 control and 6 KO animals across 5 litters. **(F–H)** MIPs from laser scanning confocal z-stacks of whole-mount skin immunostained with antibodies against LYVE-1 and smooth muscle actin (SMA) showing aberrant SMA+ recruitment to lymphatic vessels and single SMA+ cells (*arrows*) in the interstitium in the IFT20 KO **(G,H)**. **(H_1_)** Single 1 μm z-slice. Scale bars = 50 μm. ^∗∗^*p* < 0.005; ^∗^*p* < 0.05.

**FIGURE 4:**
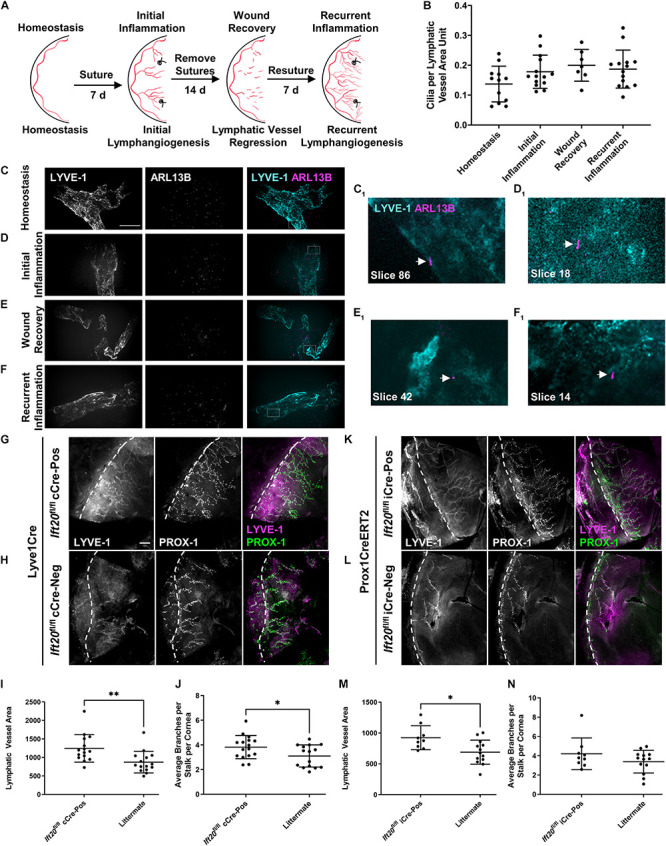
PC assemble on corneal LECs and are differentially expressed depending on inflammatory status, and IFT20 KO exacerbates corneal lymphangiogenesis. **(A)** Sequential suturing, suture removal, and resuturing in the adult mouse cornea provides an animal model to study acute and recurrent inflammation-induced lymphangiogenesis and lymphatic vessel regression during wound recovery. **(B)** Quantifications of panels **(C–F)** where each data point represents a per animal average, where “LV area unit” represents 75 μm^2^ of lymphatic vessel. Corneas under the following inflammatory microenvironments were immunostained with antibodies against ARL13B, acetylated α-tubulin (not shown), and LYVE-1: **(C)** homeostasis, **(D)** initial inflammation, **(E)** wound recovery, and **(F)** recurrent inflammation. **(C–F)** MIPs from spinning disk confocal z-stacks of whole mount corneas demonstrate the presence of primary cilia on corneal LECs during all phases of the model with a trend to increase following activation of lymphatic vessels by inflammation. (**C_1_–F_1_**) Arrows indicate abluminal primary cilia on LECs in a single 0.15 μm z-slice. For each condition, 2–5 fields of view were analyzed from each of at least seven mice across four experiments. Scale bar = 50 μm. **(G–N)** Initial corneal lymphangiogenesis was stimulated by placing sutures in each quadrant of the cornea of Lyve1Cre;*Ift20*^*fl/fl*^ **(G–J)** or Prox1CreERT2;*Ift20*^*fl/fl*^ **(K–N)** mice and littermate controls for 7 days. Littermates of all intermediate genotypes including Cre-negative and *Ift20*^*fl/+*^ were included. Corneas were harvested and immunostained with antibodies against LYVE-1 and PROX-1 and imaged by epifluorescence microscopy. **(I,J,M,N)** Quantifications of **(G,H,K,L)** where each data point represents a per animal average from fields of view encompassing the entire cornea. Scale bar = 200 μm. cCre = constitutive Cre. iCre = inducible Cre. Dashed lines indicate limbus. ^∗∗^*p* < 0.005; ^∗^*p* < 0.05.

Primary cilia *in vitro* were counted manually using the cell counter plugin in Fiji (NIH ImageJ) (Figure 1E). Lymphatic vessel densities were quantified by manually outlining lymphatic vessel area on maximum intensity projections (MIPs) using the freehand selections plugin in Fiji (Figure 2G), manual object identification in CellProfiler (Figure 3E), or by overlaying a grid onto a MIP and counting grid squares in Fiji (Figures 4B,I,M and Supplementary Figure 6C). Lymphatic vessel diameters, junctions, and branches were measured and counted manually using the segmented lines plugin and cell counter plugin, respectively, in Fiji (Figures 2H–J, 4J,N and Supplementary Figure 6D). Lymphatic vessel area filled with red blood cells was determined by dividing lymphatic vessel area containing red blood cells by total lymphatic vessel area per field of view from MIPs. Both of these areas were determined by manual object identification in CellProfiler (Figure 3E). Blood vessel junctions and branches per junction were counted using the cell counter plugin in Fiji while scrolling through a z-stack (Figure 3C). Primary cilia on LECs *in vivo* were identified by trimming confocal z-stacks to remove z-slices above and below lymphatic vasculature. Primary cilia on trimmed MIPs were then counted by automated primary object identification in CellProfiler. The primary cilia count was then masked with manually identified lymphatic vessel area in CellProfiler to identify primary cilia on LECs (Figure 2B). Total primary cilia on full stack MIPs were quantified by automated primary object identification in CellProfiler and related to the tissue volume imaged (Figure 2B), where one volume unit was set to 300 μm^3^. For primary cilia per lymphatic vessel area unit, the lymphatic vessel area was multiplied by two to account for the top and bottom surfaces of the vessel. The data is presented as primary cilia per lymphatic vessel area unit, where one area unit was defined as 75 μm^2^ (Figure 2B). Primary cilia on LECs *in vivo* were also identified manually using the cell counter plugin in Fiji by identifying primary cilia projecting from lymphatic endothelial cells while scrolling through confocal z-stacks (Figure 4B). The lymphatic vessel area was multiplied by two and one unit set equal to 75 μm^2^ as above. Percent ciliated LECs was determined by counting PROX-1+ nuclei in confocal z-stacks and identifying primary cilia projecting from each individual LEC, also expressing tdTomato (Supplementary Figure 5B). Percent proliferating LECs in E16.5 skin was determined using the cell counter plugin in Fiji to identify PROX-1+ and PROX-1/pHH3 double positive LECs in confocal z-stacks (Supplementary Figure 4C). Percent proliferating LECs in adult cornea was quantified using automated primary object identification in CellProfiler. PROX-1+ LECs were masked with pHH3+ nuclei to determine the number of proliferating LECs (Supplementary Figures 4D,E). Image brightness and contrast were adjusted in some cases to improve signal/noise for visualization in thick tissue samples.

### Western Blotting

Immortalized mouse mesenteric lymphatic endothelial cells from confluent 9.6 cm^2^ wells (six-well plates) were lysed using RIPA buffer (Thermo Fisher Scientific, 89900) plus 1X Halt protease and phosphatase inhibitors with EDTA (Thermo Fisher Scientific) and cleared by centrifugation. Cell lysates were subjected to Western blot analysis using the XCell II^TM^ Blot Module system (Invitrogen, EI9051). PVDF membranes were probed with primary antibodies against PROX-1 and β-actin as a loading control followed by Licor IRDye secondary antibodies. Membranes were imaged using a LI-COR Odyssey CLx imager with near-infrared Western blot detection and analyzed using ImageStudioLite software.

### Statistics

Data were analyzed using GraphPad Prism 8 and 9 software (GraphPad Software, Inc.) using Student’s or Welch’s T-Test or One-Way ANOVA with Dunnett’s Multiple Comparisons Post-Test. *p* values and replicates are indicated in figure legends. Error bars show mean and standard deviation.

## Results

### Primary Cilia Assemble on Lymphatic Endothelial Cells *in vitro* and *in vivo*

Our first goal was to definitively identify the presence or absence of primary cilia on LECs in culture and in mouse tissue. Using immunofluorescence microscopy, we identified ARL13B+ primary cilia on LECs *in vitro* on both SVLECs (Figure 1A) and primary adult HDLECs (Figure 1B). We confirmed that SVLECs express the master regulator LEC transcription factor PROX-1 by Western blot and form tubes in an *in vitro* tubulogenesis assay (Supplementary Figure 1). Immunofluorescence staining confirmed expression of IFT20 in SVLECs and HDLECs and marked the position of the Golgi apparatus. Approximately 60% of SVLECs displayed primary cilia under serum starvation, while approximately 18% were ciliated in complete media (Figure 1E). This is consistent with findings in other cell types in which serum starvation causes cell cycle arrest, thus enabling primary cilium assembly. Immunofluorescence staining of adult C57BL/6 ear skin revealed primary cilia present on LYVE-1+ lymphatic vessels in homeostasis (Figures 1C,D). Analysis of single 1 μm z-slices from laser scanning confocal z-stacks showed the presence of primary cilia on both the abluminal (Figures 1C1,D2, slices 10, 26) and luminal (Figures 1D1,D2, slice 18) domains of lymphatic vessels. The intraluminal space was identified by the presence of integrin-α9+ lymphatic valves (Bazigou et al., 2009; Figure 1D). Our analysis did not directly interrogate or identify primary cilia on mature valve LECs, rather valves were used to identify intraluminal z-planes. A 3D surface rendering of LYVE-1 and ARL13B signals confirmed the presence of cilia projecting both out toward the interstitium and into the lymphatic vessel lumen (Supplementary Figure 2). Analysis of E16.5 mouse mesenteric lymphatic vessels also showed both luminal and abluminal primary cilia, demonstrating conservation of ciliation during development of mesenteric lymphatic vessels, which form through aggregation of LEC progenitors and lymphvasculogenesis rather than sprouting from embryonic veins (Stanczuk et al., 2015; Supplementary Figure 3). Our *in vitro* experiments demonstrate that primary cilia on LECs adopt the expected perinuclear cellular localization and can be induced by serum starvation. These are conserved features of primary cilia *in vitro* across many cell types and indicate that regulation of ciliary dynamics is likely similar in LECs. Our *in vivo* data show that primary cilia are present on luminal and abluminal domains of LECs organized into functional vessels in mammalian tissue, suggesting that they may perform domain-specific functions.

### IFT20 KO Causes Lymphatic Vessel Patterning Defects During Mammalian Development

Given that we had now identified primary cilia on mouse and human LECs *in vitro* and on mouse lymphatic vessels *in vivo*, we next wanted to know if primary cilia are important for lymphatic vessel patterning during mouse development. To disrupt primary cilia *in vivo*, we developed a tamoxifen-inducible global IFT20 KO mouse model (*Ift20*^*fl/fl*^;CAGG-CreER^TM^). IFT20 is required for primary cilia assembly and traffic of cargo, including signaling receptors, from the Golgi to the cilium and up its length via anterograde intraflagellar transport as part of the IFT-B complex (Follit et al., 2006, 2008; Schmid et al., 2018). We induced deletion of IFT20 at E7.5, just before LECs begin sprouting from the cardinal vein, and harvested embryos at E16.5, when dorsal lymphatic vessels have come together at the midline and mesenteric lymphatics are beginning to form valves. Though extraciliary functions of IFT20 have been identified (Finetti et al., 2009, 2020), IFT20 KO embryos phenocopied common developmental defects of other ciliopathy models, including hydrops/edema, polydactyly, and craniofacial abnormalities (Figure 2A). A subset of KO embryos also displayed gross hemorrhage and vascular ballooning (Figure 2A, right panel). As expected, primary cilia incidence was reduced both in the skin overall and on LECs specifically (Figure 2B). Embryonic edema has been attributed to cardiac defects in some mouse models. Other models, such as Prox1CreERT2;*Cdh5*^*fl/fl*^ and *Pkd1*^–/–^, demonstrate normal heart development and attribute edema directly to lymphatic malfunction (Outeda et al., 2014; Hägerling et al., 2018). In addition to edema, our IFT20 KO model recapitulated common lymphatic developmental defects, such as dilated and variable lumen size, disrupted branching, and intraluminal red blood cells (Srinivasan and Oliver, 2011). Two photon microscopy of whole-mount embryonic skin immunostained for LYVE-1, PROX-1, and NRP-2 revealed malformed lymphatic vasculature with increased lymphatic vessel density and branching vs. littermate controls (Figures 2C–L). Lymphatic vessel diameter was increased in both overall caliber and range, with areas of either significant dilation or constriction evident in KO samples. Number of vessels meeting at junctions was increased in KOs, with nearly 40% of junctions composed of four or more branches. Number of junctions per field of view did not change. LEC proliferation, as measured by colocalization of phosphorylated histone H3 and PROX-1, trended to increase in KO vs. control but was not statistically significant (Supplementary Figures 4A–C). This result correlates with the increased lymphatic vessel density and junction complexity, but indicates that dramatically increased proliferation is not a feature of the IFT20 KO lymphatic vasculature at this time point during development. Red blood cells were also visualized inside lymphatic vessel lumens (Figure 2D, arrows). Formation of lymphatic vessel valves is not complete until approximately E18 (Koltowska et al., 2013), thus we were unable to evaluate valve formation in these E16.5 skin samples. Our data demonstrate that IFT20 KO reduces the prevalence of primary cilia overall and on LECs, induces common ciliopathy phenotypes, and dysregulates lymphatic vessel patterning.

### Lymphatic Vessels Are Filled With Red Blood Cells and Recruit Smooth Muscle Actin+ Cells in IFT20 KO Embryos

Our analysis of E16.5 IFT20 KO dorsal skin identified LYVE-1/PROX-1+ lymphatic vessels with autofluorescent intraluminal red blood cells (RBCs; Figure 2D, arrows) as well as extravascular RBCs. The presence of RBCs in lymphatic vessels is a feature of many developmental lymphangiogenesis mutants and has been attributed to failure to adequately separate blood and lymphatic vasculatures due to lymphovenous valve insufficiency, to improper blood-lymphatic vessel anastomoses, or to clearing of extravascular RBCs from the interstitium following blood vessel hemorrhage. To visualize blood and lymphatic vasculature together, we immunostained skin for LYVE-1, PROX-1, and podocalyxin. Podocalyxin is a sialoglycoprotein that is expressed by both blood and lymphatic endothelial cells. This staining strategy resulted in lymphatic vessels labeled with all three markers and blood vessels labeled with podocalyxin only (Figures 3A,B). Blood vessel branching was dysregulated in IFT20 KOs (Figure 3C). Blood vessel junctions per field of view were decreased approximately by half in KO vs. control, while number of junctions composed of four or more branches was increased to more than 15%. As expected RBCs were restricted to blood vessel lumens in control embryo samples. In contrast, RBCs were present both free in the interstitium and within the lumen of lymphatic vessels in IFT20 KO skin. RBCs tightly packed the lumen of some sections of lymphatic vessels, while in areas of more uniform lumen caliber RBCs were more sparsely distributed, suggesting flow (Figure 3D). Overall, on average 30% of lymphatic vessels by area were filled with RBCs across six IFT20 KO embryos, ranging up to more than 70% (Figure 3E).

Aberrant recruitment of smooth muscle cells to lymphatic vessels is a feature of other models of vascular malformation. Blood vessels are invested with vascular smooth muscle cells to increase structural integrity and reduce hemorrhage. Lymphatic vessels are exposed to much lower intraluminal pressure than blood vessels. In mice, lymphatic collecting vessels are not supported by lymphatic smooth muscle cells until P14, and lymphatic capillaries lack mural cell coverage entirely. To determine if smooth muscle cell localization is disrupted by loss of primary cilia, we immunostained for smooth muscle actin (SMA) and LYVE-1. As expected, SMA+ cells in control skin smoothly covered LYVE-1-negative blood vessels (Figure 3F). In IFT20 KO skin, SMA+ cells with numerous filopodia were aberrantly recruited to lymphatic vessels (Figure 3G), covering large patches in some cases (Figure 3H). Single SMA+ cells not associated with vessels were also present in KO skin (Figure 3G, arrows).

In summary, CAGG-CreER^TM^ -mediated loss of IFT20 disrupts not only lymphatic but also blood vessel patterning and integrity. Blood vessel branching overall was reduced, while complexity at individual junctions was increased. Free RBCs and RBCs in the lymphatic vessel lumen suggest compromised blood vessel integrity, which may be explained in part by mislocalization of SMA+ cells individually in the interstitium and to lymphatic vessels.

### Primary Cilia Assemble on Corneal Lymphatic Vessels During Acute, Recurrent, and Tumor-Associated Inflammation and Wound Healing, and IFT20 KO Exacerbates Corneal Lymphangiogenesis in a LEC-Autonomous Manner During Acute Inflammation

To determine if primary cilia on LECs regulate lymphatic patterning in settings beyond development, we quantified primary cilia on corneal lymphatic vessels during distinct pathological conditions. We used a well-characterized corneal model of inflammation, wound healing, and recurrent inflammation to induce three diverse corneal lymphatic patterning events: initial lymphangiogenesis, lymphatic vessel regression, and recurrent lymphangiogenesis (Kelley et al., 2011, 2013; Figure 4A). In this model, placement of sutures in the cornea induces inflammation and activates LECs restricted to the limbus vasculature during homeostasis to sprout, proliferate, and migrate to expand the lymphatic vasculature into the corneal parenchyma. This initial lymphangiogenesis is dependent on VEGF-C. Removing sutures promotes resolution of inflammation, during which lymphatic vessels regress by unknown mechanisms. Resuturing causes recurrent lymphangiogenesis, also termed lymphatic vessel memory (Kelley et al., 2013), in which regressed lymphatic vessel fragments reorganize independent of VEGF-C to form a complex, robust lymphatic network. Primary cilia were present on corneal lymphatic vessels during all phases of this dynamic tissue injury model (Figures 4B–F). Stimulation of LECs to undergo lymphangiogenesis trended to increase cilia incidence in all subsequent phases of the model vs. homeostasis, though this was not statistically significant. Expression of primary cilia across diverse inflammation states suggests that primary cilia-dependent signaling may influence lymphatic patterning by complementing known mechanisms of lymphangiogenesis regulation or by supporting novel mechanisms.

We next wanted to determine if primary cilia on LECs could cell-autonomously regulate lymphatic vessel patterning. We used lymphatic-specific constitutive and tamoxifen-inducible Cres to disrupt IFT20 in adult mouse LECs and studied corneal lymphangiogenesis during initial inflammation using the model described above (Figure 4A). IFT20 KO driven by constitutive Lyve1Cre or tamoxifen-inducible Prox1CreERT2 increased new lymphatic vessel growth toward corneal sutures. (Figures 4G–L). At harvest on day 7, lymphatic vessel density was increased in *Ift20*^*fl/fl*^;Cre-positive mice vs. littermate controls in both models (Figures 4I,M). Branching was also significantly increased in the constitutive Cre model (Figures 4J,N). In some animals, a large number of very short branches sprouting from stalks was observed (Figure 4G). This together with our finding of highly ciliated tip cells (Supplementary Figure 5) indicates that cilia may be especially important for regulation of tip cell identity and function. LEC proliferation was not significantly increased at this time point during initial inflammation in either model (Supplementary Figures 4D–I). We also performed these experiments in Lyve1CreERT2;*Ift20*^*fl/fl*^ mice, but increased lymphangiogenesis in that model did not reach statistical significance (Supplementary Figure 6). These results demonstrate LEC-autonomous regulation of inflammation-associated corneal lymphangiogenesis by primary cilia.

We then turned to a mouse melanoma model to study tumor-associated corneal lymphangiogenesis. To monitor lymphatic vessel growth toward tumors, we performed these experiments in Lyve1CreERT2;tdTomato mice in which LECs express tdTomato fluorescent reporter protein. We sutured mouse corneas, injected B16-F10 cells into a corneal micropocket, and allowed tumor-associated lymphangiogenesis to proceed for 8 days (Supplementary Figure 5A). We immunostained corneas for acetylated α-tubulin and PROX-1. Primary cilia and a subset of corneal nerves expressed acetylated α-tubulin. We quantified the incidence of primary cilia on populations of lymphatic endothelial cells expected to perform different functions based on their localization within the vascular network: limbus cells, stalk/branch cells, and tip cells. LECs residing in limbus lymphatic vessels are mostly quiescent during later stages of corneal lymphatic vessel remodeling but, as the source of nascent lymphatic vessels, can be stimulated to sprout and proliferate. LECs residing in a stalk connected to the limbus or in a branch emanating from a stalk proliferate along the long axis of the vessel to increase its length. Tip cells are typically non-proliferative and provide directionality to vascular expansion through signal gradient detection, filopodia extension, and chemotaxis (Connor et al., 2016; Geng et al., 2020). We identified primary cilia on limbus, stalk/branch, and tip LECs in this model (Supplementary Figures 5B–E). Approximately 40% of limbus and stalk/branch LECs and nearly 80% of tip cells were ciliated. In addition to likely functions in chemosensing, LEC primary cilia in the tumor microenvironment may be responsible for sensing changes in tissue stiffness or regulate properties of lymphatic vessels relevant to tumor metastasis such as intercellular junctions.

These data show that primary cilia are present on corneal lymphatic vessels under a wide variety of microenvironmental conditions. LECs residing in distinct functional domains of lymphatic vessels (limbus, stalk/branch, tip) assemble primary cilia. Tip cells especially may employ primary cilia-dependent signaling pathways during lymphangiogenesis. Taken together these data suggest that ciliary signaling may be important for both maintenance of lymphatic vessels during homeostasis and during diverse lymphatic vessel remodeling events that characterize inflammation, wound healing, recurrent inflammation, and the tumor microenvironment.

## Discussion

Despite swift progress in the field of lymphatic vessel biology since the identification of lymphatic-specific markers around the turn of the century, the mechanisms regulating lymphatic vessel patterning in some physiological settings remain unclear. In this work we demonstrate that LECs possess primary cilia both *in vitro* and *in vivo* and that disruption of *Ift20*, a critical component of the intraflagellar transport machinery and a required element for ciliary assembly, results in profound lymphatic vessel patterning defects during both mammalian development and inflammation. While the developmental and inflammation-associated lymphangiogenesis phenotypes we observed in our models were not identical, they were complimentary in that lymphatic vessel density and complexity of branching were increased in both conditions and within the spectrum of phenotypes reported in other studies of dysregulation of embryonic or corneal lymphangiogenesis. This suggests that signaling through primary cilia governs cellular LEC activities important for lymphangiogenesis, such as proliferation, chemotaxis, lumen formation, shear flow sensing, and intercellular junction organization.

### Primary Cilia on Lymphatic Endothelial Cells

The diversity of microenvironments and vessel domains in which primary cilia are present on LECs implicates them broadly in lymphatic vascular biology. While primary cilia assemble on LECs *in vitro* as well as *in vivo* in development and in inflammatory and tumor microenvironments, the functioning primary cilia-dependent signaling and sensory mechanisms may be unique in each condition. We demonstrate that primary cilia are present on both the abluminal and luminal surfaces of adult and embryonic lymphatic vessels, including near lymphatic valves, though we did not directly visualize primary cilia on valve LECs. In other lumenized structures such as kidney tubules and blood vessels, primary cilia predominantly project into the lumen, serving as flow sensors (Nauli et al., 2008; AbouAlaiwi et al., 2009; Goetz et al., 2014), including regulation of vascular regression in retina (Vion et al., 2018). Intraluminal cilia in blood vessels have recently been implicated in regulation of vascular integrity independent of flow (Eisa-Beygi et al., 2018). Thus, primary cilia within the lymphatic vessel lumen may complement known mechanisms of shear stress sensing or perform flow-independent functions and may regulate lymphatic vessel regression. Abluminal cilia may be involved in direct interactions with extracellular matrix or other cells in the interstitium, such as immune cells and fibroblasts, and may also play a more prominent role in receptor-ligand mediated signaling or in sensing other microenvironmental attributes such as matrix stiffness (Frye et al., 2018). We also show that primary cilia are present on regions of corneal lymphatic vessels with distinct functional roles during lymphangiogenesis. Primary cilia are present on LECs residing in the quiescent limbus, proliferating stalk/branch, and chemosensing tip of nascent lymphatic vessels. Primary cilia localized to discrete vessel domains may themselves be specialized to regulate specific aspects of a coordinated lymphangiogenesis response, such as proliferation vs. gradient sensing. LEC-specific IFT20 KO exacerbated inflammation-associated lymphangiogenesis, strongly influencing overall lymphatic vessel density as well as branching in some specimens. While key aspects of developmental and initial inflammation-associated lymphangiogenesis are clearly regulated by canonical VEGF-C/VEGFR-2/3 mechanisms, our data provide evidence for additional complexity that may complement, augment, oppose, or modify this pathway through primary cilia-dependent mechanisms. We also demonstrate the presence of primary cilia on LECs during lymphatic vessel regression, recurrent lymphangiogenesis, and tumor-associated lymphangiogenesis, three mechanistically distinct, poorly understood lymphatic remodeling events with significant implications for wound healing, chronic inflammation, and metastasis. Thus, elucidation of primary cilia-dependent signaling mechanisms on LECs under these conditions may provide new targets for therapeutic modulation of disease. Taken together, LEC primary cilia are likely an important feature of the lymphatic vascular signaling machinery with diverse functions depending on the microenvironment, activation state, and vascular domain of the LEC on which they reside.

### Primary Cilia in Lymphatic Vessel Development

Our IFT20 KO model demonstrates defective lymphatic vessel patterning during development. Similar developmental lymphatic abnormalities occurred when pathways known to function via primary cilia in other cell types were disrupted globally or in LECs. For example, PKD1/2 KO mice displayed edema, dilated lymphatics, and altered lymphatic branching. This was attributed to failed LEC polarity and disorganization of actin stress fibers and VE-cadherin junctions (Coxam et al., 2014; Outeda et al., 2014; Huang et al., 2015). Similarly, loss of TGFBR1/2 reduced LEC filopodia formation, disrupted branching, and caused lymphatic hyperplasia and edema (James et al., 2013). *Fat4* was recently identified as a LEC target gene of transcription factor GATA2, which regulates many aspects of lymphatic specification and maturation. FAT4 is an atypical cadherin that localizes to primary cilia and regulates planar cell polarity (Saburi et al., 2008). FAT4 KO during developmental lymphangiogenesis caused edema and defects in vessel caliber and branching due to disrupted LEC polarity (Betterman et al., 2020). Furthermore, LEC-specific KO of NOTCH1 caused edema and lymphatic hyperplasia, including increased proliferation, sprouting, and junction reorganization (Murtomaki et al., 2013; Fatima et al., 2014). These studies together with our data suggest that the primary cilium may function as a hub for these signaling mechanisms in LECs as in other cell types.

Human primary lymphedema may also be linked to dysregulation of primary cilia function. HKLLS1 (Hennekam lymphangiectasia-lymphedema syndrome 1, OMIM #235510) can be caused by mutation of *FAT4*, and in addition to lymphedema may also present with camptodactyly or syndactyly (Alders et al., 2014), digit aberrations common in ciliopathies (Baker and Beales, 2009; Norris and Grimes, 2012). Similarly, mutation of *KIF11*, a microtubule-based motor protein, results in MLCRD (microcephaly with or without chorioretinopathy, lymphedema, or mental retardation, OMIM #152950) syndrome (Ostergaard et al., 2012). KIF11 has recently been shown to regulate primary cilia assembly dynamics (Zalenski et al., 2020) and been implicated in human retinal ciliopathy (Birtel et al., 2017). Future work is needed to determine how LEC primary cilia may influence the pathogenesis of human disease.

### Vascular Integrity and Flow Sensing

Two striking features of E16.5 IFT20 KO skin are blood-filled lymphatic vessels and aberrant localization of SMA+ cells singly within the interstitium and on lymphatic vessels. Lymphovenous valves form at the interface of blood and lymphatic vasculatures during expansion of the first lymphatic capillaries following upregulation of PROX-1 in venous endothelial cells. Blood-lymph separation is then reinforced by interactions between podoplanin on LECs and CLEC receptors on platelets (Bertozzi et al., 2010; Hess et al., 2013). Lymphovenous valve insufficiency is a feature of several lymphedema mouse models with disruptions in genes including *Prox1, Foxc2, Cx37, Gata2, Pdpn*, or *Clec2* (Uhrin et al., 2010; Hess et al., 2013; Geng et al., 2016; Bianchi et al., 2018). The extent of blood-filled lymphatics in our model suggests that lymphovenous valve insufficiency may be a consequence of loss of primary cilia. In blood endothelium, primary cilia are important for maintaining blood vessel integrity in an endothelial cell-autonomous manner (AbouAlaiwi et al., 2009; Kallakuri et al., 2015; Eisa-Beygi et al., 2018; Liu et al., 2018). Our data also support this model, as our analysis revealed widespread extravascular RBCs in IFT20 KO skin. Blood vessel integrity may also have been compromised due to recruitment of SMA+ cells to dilated lymphatic vessels in embryonic skin. Aberrant localization of SMA+ cells on lymphatic vessels has been linked to defects in lymph flow and flow sensing in developmental lymphangiogenesis mutants including *Foxc2^–/–^, Ang2^–/–^*, and *Clec2*^–/–^(Petrova et al., 2004; Dellinger et al., 2008; Sweet et al., 2015), though we cannot assign a LEC-autonomous recruitment mechanism in our model because of our use of the global Cre deleter. It is possible that changes in sensing external forces, such as vessel dilation leading to endothelial stretch (Stratman et al., 2020) or reduced fluid shear stress sensing could induce chemoattractant expression to recruit SMCs. For example, *Foxc2*^–/–^ mouse embryos showed dilated lymphatic vessels with associated SMA+ cells. FOXC2 is a multifunctional flow-dependent transcription factor (Sabine et al., 2015; Norden et al., 2020) and negative regulator of collagen IV production and PDGF-B secretion, which recruits SMCs. Thus, loss of FOXC2 upregulated PDGF-B and collagen IV and recruited mural cells to mutant dermal lymphatic vessels (Petrova et al., 2004). Future studies should determine the role of LEC primary cilia in complementing known mechanisms of fluid shear stress sensing and mechanotrasduction (Sabine et al., 2012;2015; Gordon et al., 2020), such as primary cilium-localized mechanosensative ion channels that have been implicated in lymphatic function including PIEZO1 and TRPV4 (Lee et al., 2015; Behringer et al., 2017; Choi et al., 2019; Miyazaki et al., 2019; Solari et al., 2020), modulation of FOXC2 expression or phosphorylation (Ivanov et al., 2013; Dieterich et al., 2017), and mural cell recruitment or lack thereof.

## Conclusion

This study presents the LEC primary cilium as a novel regulator of lymphatic vessel patterning. Studying primary cilia-dependent signaling is a new approach to identify mechanisms of lymphatic vessel patterning and function not fully explained by current paradigms. Ongoing work in our laboratory seeks to elucidate the molecular mechanisms by which primary cilia-dependent signaling controls LEC activities, such as sprouting and migration, that are required for lymphatic vessel remodeling events. Studying primary cilia function on LECs is a new enterprise for the field of lymphatic vessel biology. Understanding how primary cilia sense and transmit extracellular soluble and mechanical signals to modify LEC signaling networks has the potential to elucidate fundamental aspects of LEC biology and may identify new targetable mechanisms of lymphatic vessel regulation.

## Data Availability Statement

The raw data supporting the conclusions of this article will be made available by the authors, without undue reservation.

## Ethics Statement

The animal study was reviewed and approved by Institutional Animal Care and Use Committees of South Dakota State University or the University of Massachusetts Medical School.

## Author Contributions

DF and GP designed the study and wrote the manuscript. DF, RH, and GP generated mice. All authors designed and performed experiments and reviewed the manuscript.

## Conflict of Interest

The authors declare that the research was conducted in the absence of any commercial or financial relationships that could be construed as a potential conflict of interest.

## Abbreviations

FOV: field of view
LEC: lymphatic endothelial cell
MIP: maximum intensity projection
RBC: red blood cell
RT: room temperature.
