# Supplementary figures and images for: Loss of Primary Cilia Protein IFT20 Dysregulates Lymphatic Vessel Patterning in Development and Inflammation
Source: Front Cell Dev Biol. 2021 May 14;9:672625. doi: 10.3389/fcell.2021.672625 (PMC8160126; doi:10.3389/fcell.2021.672625)

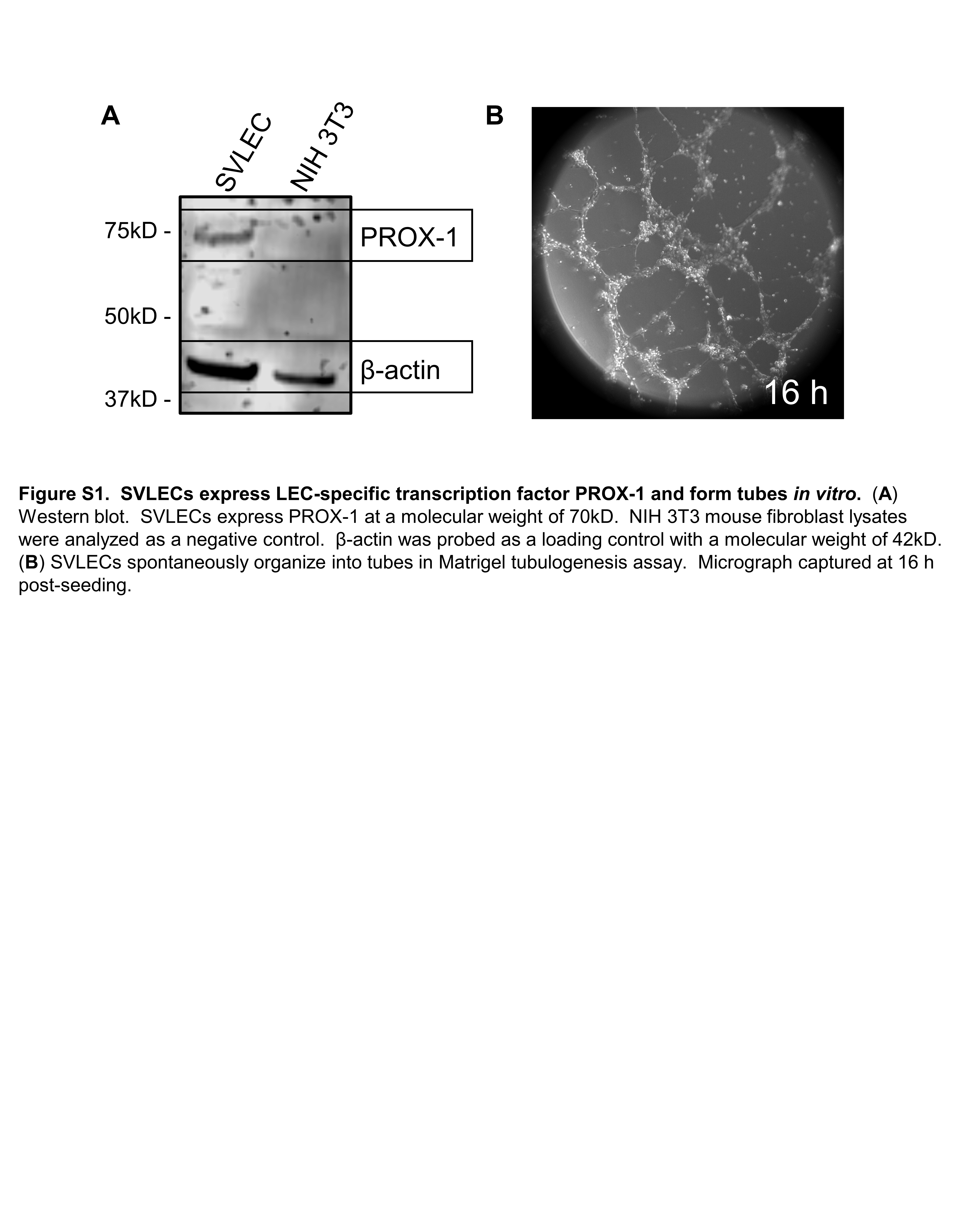

Supplement: Supplementary file 1 [file Image_1.TIF]

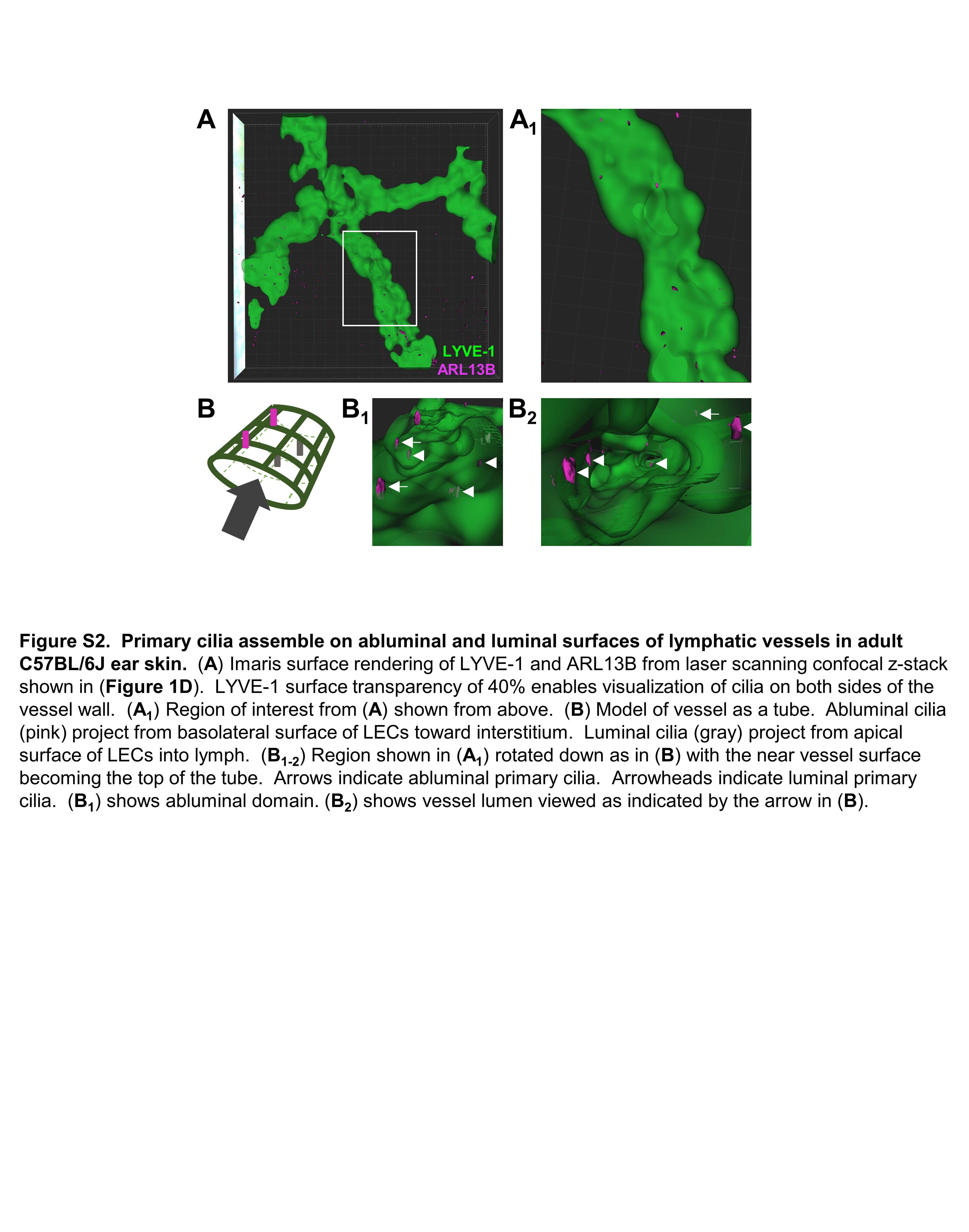

Supplement: Supplementary file 2 [file Image_2.TIF]

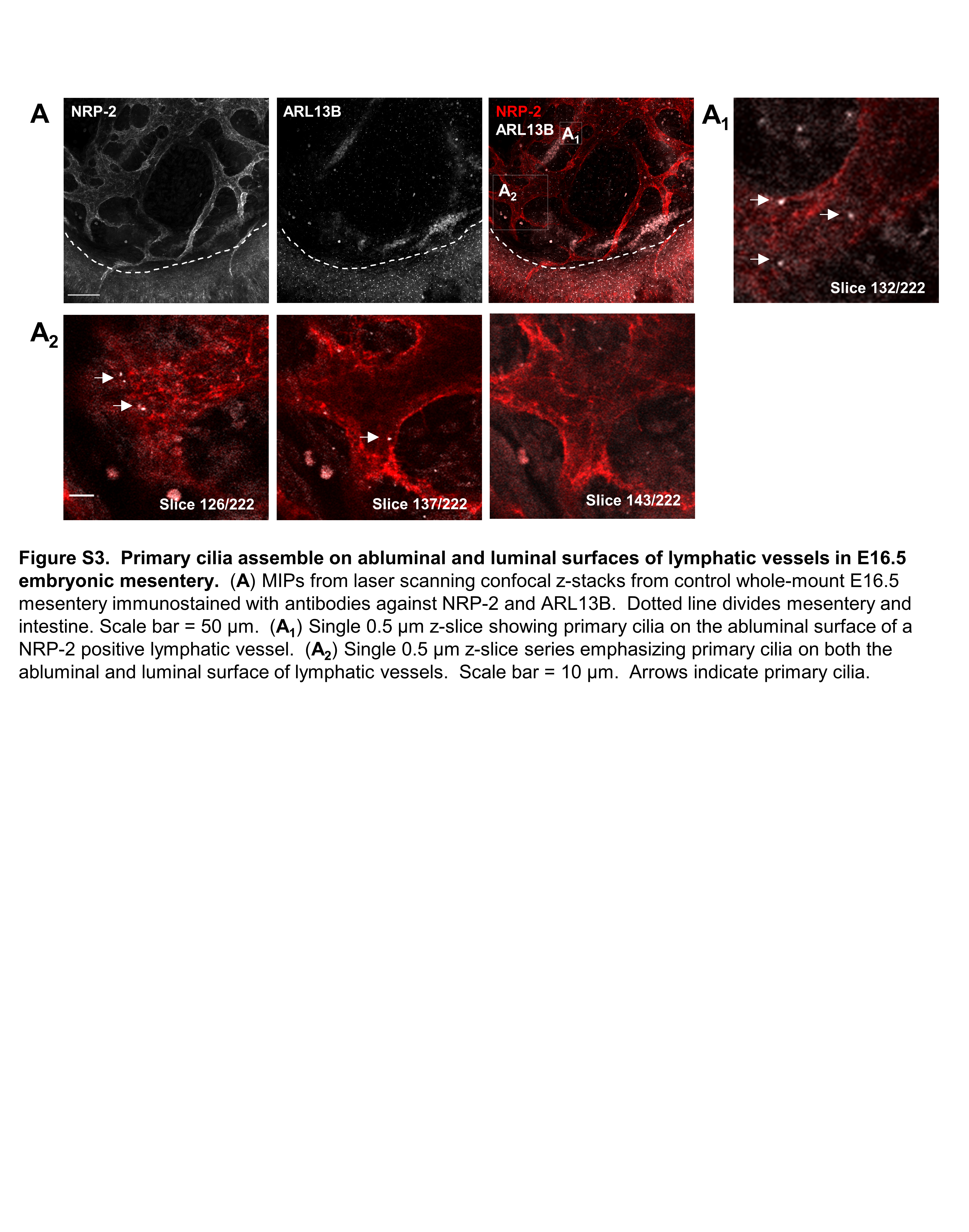

Supplement: Supplementary file 3 [file Image_3.TIF]

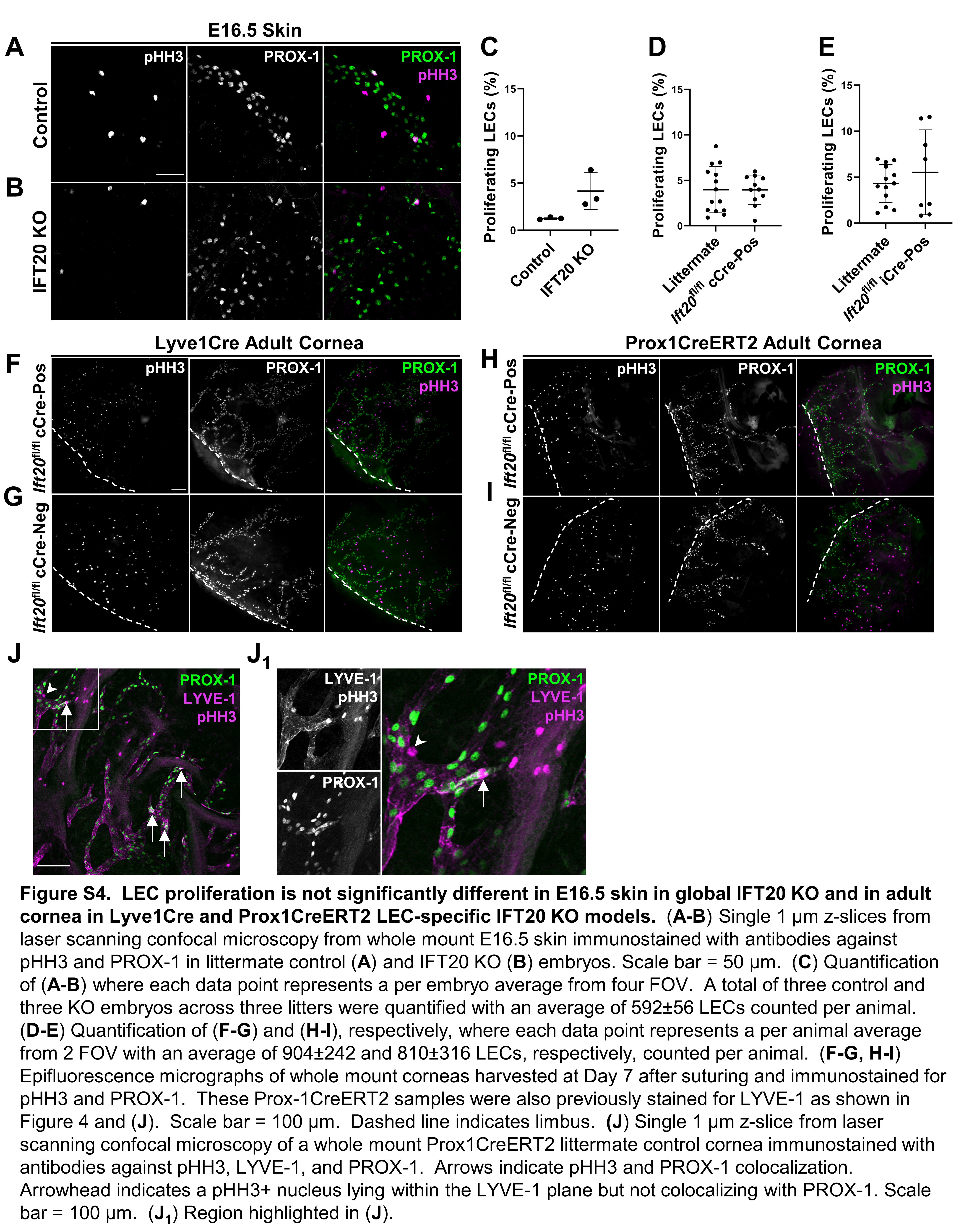

Supplement: Supplementary file 4 [file Image_4.TIF]

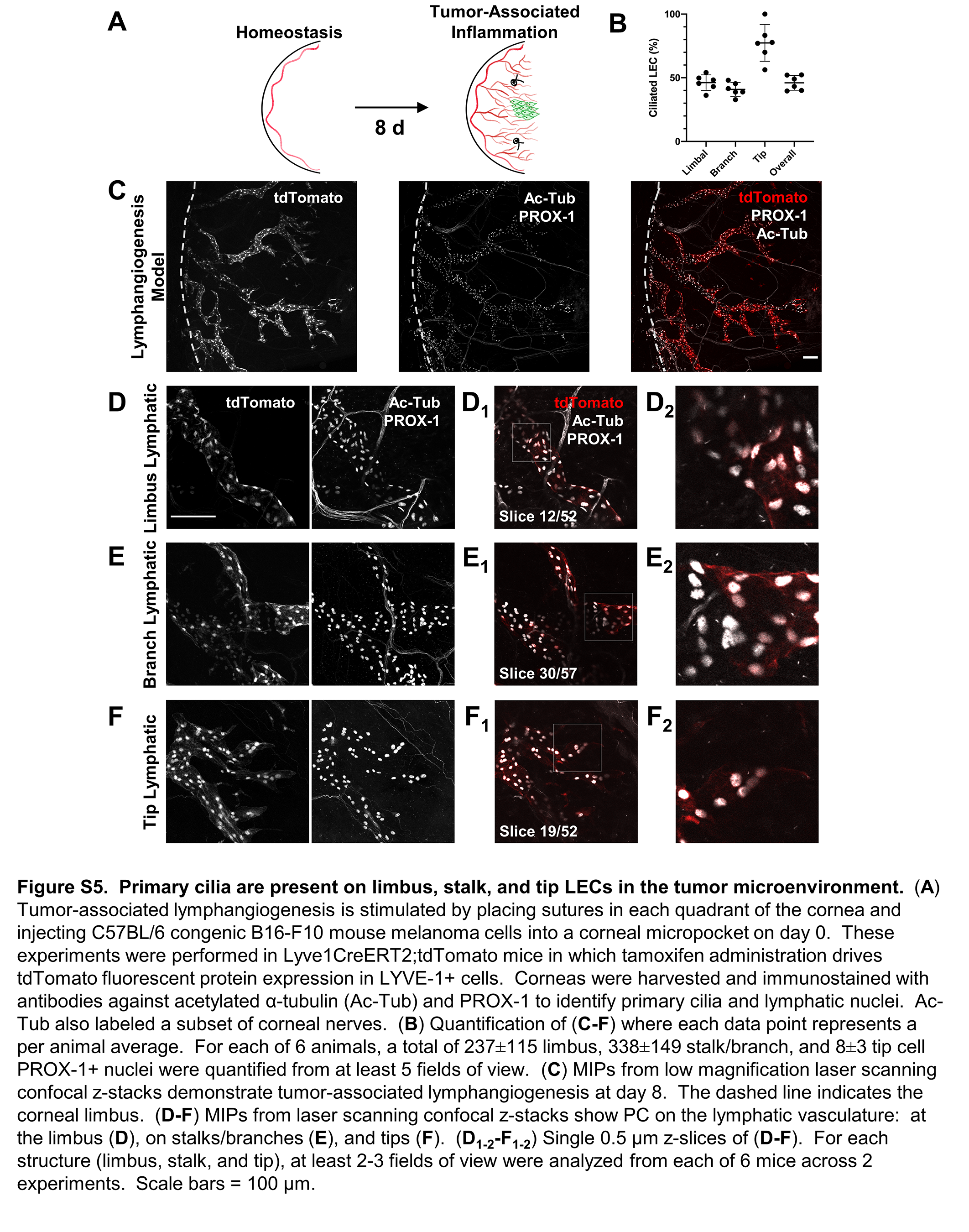

Supplement: Supplementary file 5 [file Image_5.TIF]

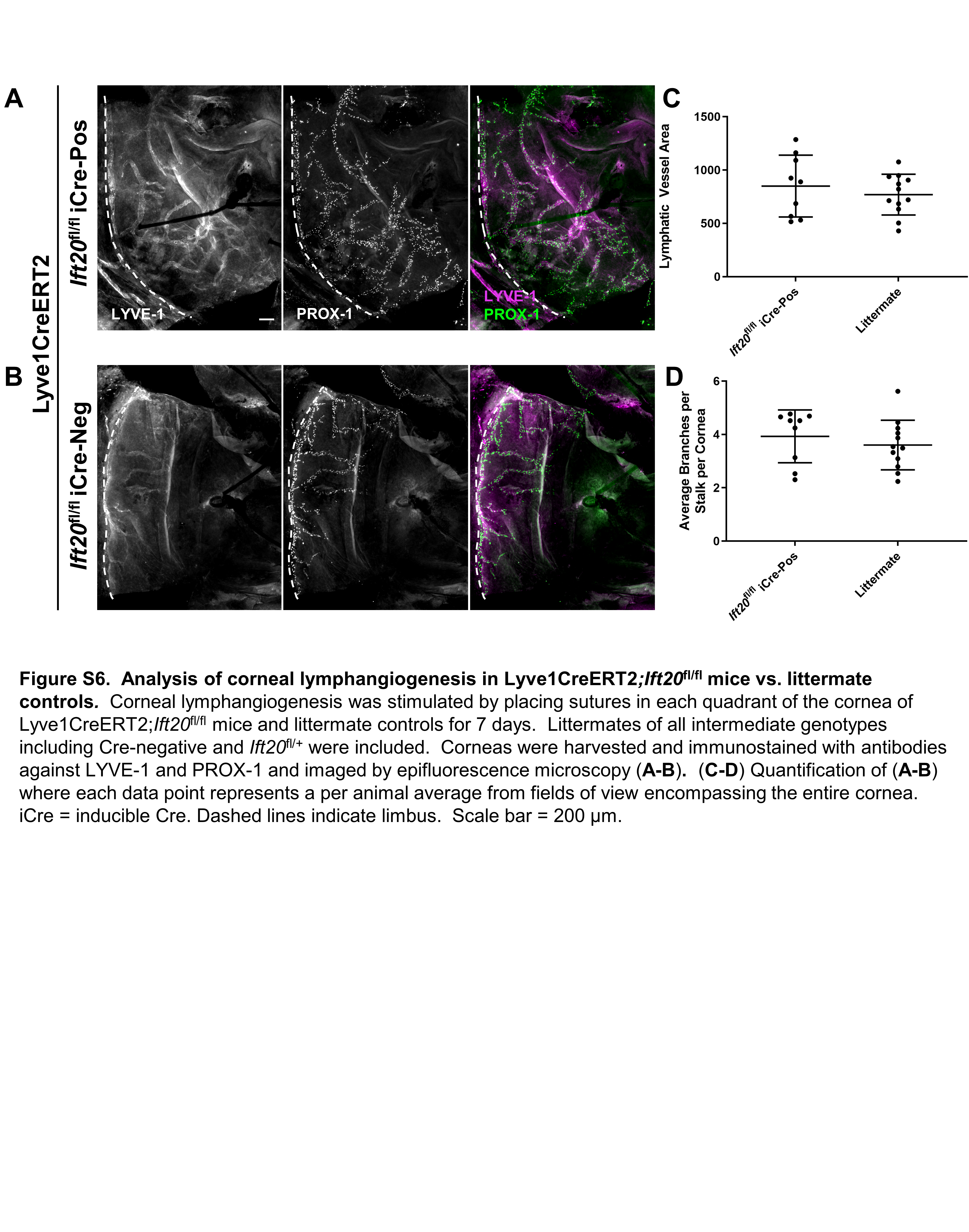

Supplement: Supplementary file 6 [file Image_6.TIF]
